# Obituary

**Published:** 1885-02

**Authors:** 


### OBITUARY.

Died, at his residence in Simcoe, Ontario, November 26th, 1884, Lyman Wells, L. D. S., a dental practitioner of more than thirty years' standing. He was born at Canaan, New Hampshire, January 7th, 1809, and was of excellent lineage. His mother was a niece of James Otis, of revolutionary fame, and all his ancestors were officers, some of them of great distinction, in the revolutionary army. Mr. Wells removed to Upper Canada about the year 1835, and had for the whole, or nearly all the time since, been a resident of Simcoe, in the County of Norfolk.

Twenty-eight years ago the editor of this journal was a patient of Mr. Wells. From that time to the period of his death the acquaintance and friendship was intimate and warm, and we can personally testify to the high character, the moral worth, the professional ability, and the genial qualities of the deceased. He was one of the early experimenters in and practical workers of the art of photography, or "daguerreotyping," as it was then called. About the year 1850, his attention having been attracted to dentistry, he came to Buffalo and entered the office of Dr. Nathan Whitcomb, and after spending sufficient time to become acquainted with the art, he returned to Simcoe and opened an office, where he continued in practice up to the time of his death.

Mr. Wells was one of the charter members of the Ontario Dental Society, and early in its history served as its President. He always retained a keen interest in its welfare, although for some time his health had been such as to forbid his presence at the meetings. He was an honorary member of the Eighth District Dental Society of the State of New York, and until within a few years was usually present at its meetings. His home reputation as a dentist was very high, and until strength failed him because of his years, he enjoyed a large practice, patients visiting him from remote localities. In church, masonic, and social circles, he was always very popular, and in his latter days was revered as a father. There is many a professional friend beside the writer to whom the tidings of his death will bring a deep sorrow and lasting regret.

